# Health Related Quality of Life (HRQOL) in pediatric population after surgical intervention in traumatic lower limb injuries: A prospective cohort

**DOI:** 10.12669/pjms.39.4.7128

**Published:** 2023

**Authors:** Muhammad Mustafa Hashmi, Muhammad Rehan Akram, Fatima Naumeri, Muhammad Faizan Akram

**Affiliations:** 1 Muhammad Mustafa Hashmi, MBBS. King Edward Medical University, Lahore, Pakistan; 2 Muhammad Rehan Akram, MBBS. King Edward Medical University, Lahore, Pakistan; 3 Fatima Naumeri, MCPS, FCPS, CME. Department of Pediatric Surgery, Services Institute of Medical Sciences/Services Hospital. Lahore, Pakistan; 4 Muhammad Faizan Akram, MBSS. Pediatric and Neonatal Medicine Department, Manawan Hospital, Lahore, Pakistan

**Keywords:** Pediatric trauma, Childhood injuries, Quality of life, Patient related outcomes, Health related quality of life

## Abstract

**Objective:**

To assess health related quality of life (HRQOL) of children who had undergone surgical intervention after lower limb trauma.

**Methods:**

A prospective observational study was conducted at KEMU and UCHS, from September 2021 to June 2022. Fifty children aged 5 to 12 years who had undergone surgical intervention for lower limb trauma were included. Patients whose parents couldn’t understand Urdu or English, or with polytrauma, needing amputation and/or comorbidities were excluded. Another fifty-school going, age matched children were enrolled as controls. After informed consent, two responses from participants and parents on PedsQL questionnaires were recorded, first one week after discharge and second after six months, assessing age specific quality of life in four domains (physical, emotional, social, school). Impaired HRQOL was defined as a score that was more than 1SD below the mean of healthy sample.

**Results:**

Baseline HRQOL scores were significantly lower in patients and parents reported data taken at one week, overall difference of 39.13 in total HRQOL scores (effect size, d 2.76). Difference of 39.86 was observed between baseline and follow-up data taken from parents with the highest effect size (d=3.34) in physical functioning scores. There was a significant difference of 10.07 between the total HRQOL scores of patients on follow up and controls, and HRQOL patients’ scores were higher than controls across all domains. Parent reported scores were less than those reported by children in almost all cases.

**Conclusion:**

HRQOL of children at discharge after management of lower limb trauma was lower than age-matched control group. It, however, significantly exceeded controls’ scores after six months showing complete recovery.

## INTRODUCTION

Injuries are common in childhood, with studies showing that on average about 25% of the children are injured each year. Fractures account for 10-25% of the childhood injuries and may lead to considerable restriction of activity.^1^ A national injury survey in 1997 revealed an overall injury incidence of 41 per 1000 and a mean age of only 29 years for those injured.^2^ Specific studies have documented the burden of injury on male children under the age of 15 years and provided an estimated incidence of 19.2 per 100 child-years for non-fatal injuries.^3^ Injuries are overlooked as contributors to global inequities in health, yet the long-term disabilities they frequently produce represent a significant burden.

In addition, inadequate emergency care systems, and reduced life expectancy cause difficulties on a whole new level in low/middle-income countries (LMIC).^4^,^5^ Childhood is a critical period in growth and development, forming the basis for healthy adult life. Therefore, when debilitating physical trauma occurs, there is the potential for substantial impact across a few years if not the whole lifespan. Although mortality is a useful and easily defined parameter, it does not accurately reflect the significant burden of injury on those who survive.

Assessment of quality of life gives an insight about the efficiency, reliability and standard of care provided by the healthcare system. Patient reported outcomes (PROs) are a measurement of any aspect of a patient’s health status that comes directly from the patient which includes disease symptoms, physical functioning and quality of life (QOL).^6^ Health- related quality of life (HRQOL) is a modern tool for measuring PROs, taking into account subjective attitudes, and experiences from physical, emotional, social and cognitive domains pertaining to an individual’s health.^7^,^8^ In addition, HRQOL provides a good benchmark for assessing the benefit of surgical and other therapeutic interventions and reveals where the best patient-sensitive gains can be made.

Little work has been done to assess pediatric injuries in Pakistan and by appraising the PROs in children who have undergone surgery after trauma to the lower limb, our aim was to follow the physical recovery and quality of life to get an insight into the quality of healthcare services provided.

We hypothesized that at one week following discharge, HRQOL in pediatric population will be less than the control group and comparable to it after six months.

## METHODS

This prospective observational study was conducted in the departments of pediatric surgery, orthopedic surgery, and plastic surgery at Mayo Hospital/ King Edward Medical University (KEMU) and in the orthopedic and plastic surgery department of The Children’s Hospital/ University of Child Health Sciences (UCHS), Lahore after taking ethical approval (IRB KEMU letter no.659/RC/KEMU and UCHS letter no.537/CH-UCHS), from September 2021 to June 2022. A sample size of 90 subjects (45 in patient group and 45 in control group) was calculated by conducting a pilot study for mean HRQOL scores in patients and controls, using 5% level of significance and 90% power of test. Children between 5 to 12 years of age were included who suffered from lower limb trauma (bone or soft tissue injury due to road traffic accident, burns, fall or sports injury) and had undergone surgical intervention (debridement, irrigation, fracture management/ dislocation reduction, fasciotomy for burn or compartment syndrome, wound coverage by grafting/ flap after saline dressings/ VAC), using purposive sampling.

Whereas patients whose parents/guardians did not give informed consent, those who couldn’t understand Urdu or English, or with polytrauma or presented with gunshot wound, associated nerve and vessel injury of lower limb, needing amputation and/or patients with other comorbidities (congenital heart defects, chromosomal abnormalities, neuromuscular disorder, and psychological impairments) were excluded from the study. In control group, age-matched school going children with no comorbidities, were enrolled.

After informed consent from parents/guardians, responses of the participants and parents regarding PedsQL questionnaires were recorded either in person or on telephone depending on the feasibility of the parents. Two responses were collected from the patients and their parents in the patients’ group. First after seven days of discharge and the second six months after the first response. Response from the control group was collected only once at a local school (parents and children both). A total of 62 patients fulfilling our inclusion/exclusion criteria were enrolled in the patient group, out of which 54 responded to our first data call and then at the second data call 50 out of these 54 patients responded (participation level of 80.6%). Thus, 50 patients were enrolled in the patient group and another 50 in the control group.

### PedsQL™ Questionnaire:

Was used to assess the HRQOL. Permission was sought from Mapi Research Trust to use the questionnaires.^9^ This questionnaire has well established validity and reliability in different diseases and has been used in different populations.^10^,^11^ PedsQL 4.0 Generic Core Scale includes 23 items and assesses the quality of life in four different domains (physical, emotional, social, school) and is age specific. Each PedsQL item asks the respondent to describe how frequently the child has a problem on a Likert scale that ranges from “never” to “almost always”.

The PedsQL scales can be aggregated into summary scales of physical (same as physical functioning scale), psychosocial (emotional, social, and school functioning scales), and overall HRQOL (all four scales). Scale scores range between 0 (poorest health) and 100 (best health). Impaired HRQOL was defined as a score that was more than 1SD below the mean of healthy sample.^10^ Sociodemographic data included age, gender, socioeconomic background (World Bank’s poverty line definition of US $ ≤1.9/day) and parental education (primary, middle, higher, intermediate, graduate). Clinical data included mode and type of injury, three different injury severity scales namely pediatric trauma score (PTS), Nerve injury, ischemia, soft tissue, skeletal injury, shock, age of patient score (NISSSA), The Ganga Hospital Injury Severity Score (GHOISS) and duration of hospital stay.

Data were analyzed using the Statistical Package for Social Sciences (SPSS 27). Mean and standard deviation (SD) were calculated for continuous variables and frequencies were calculated for categorical variables. Differences in HRQOL of the baseline and follow up data of the patients were calculated using paired t-test, while the differences in HRQOL between patients and their age-matched school going children were calculated using independent sample t-test and a p value of <0.05 was taken as statistically significant. Multiple regression was used for associations of various socioeconomic and clinical predictors on HRQOL.

## RESULTS

Demographic data is shown in Table-I. The majority (n=34, 68%) of participants were from Mayo Hospital Lahore. In the patient group, 8% of all injuries were soft tissue injuries, 12% mixed injuries, 10% compartment syndrome/ dislocations/ lacerations, fractures 30%, whereas avulsions made up 40% of the total injuries. Mode was spoke wheel injuries in 46% of patients, 38% suffered road traffic accidents, 8% had blunt/sharp trauma, 6% had burns and only 2% had fall related injuries. No patient had physical deformity on follow-up. Assessment of life quality data (baseline) given by patients and their parents/ guardians and its comparison with follow-up data is given in Table-II. The only missing data was the school functioning domain in the baseline data as all the patients were not attending school owing to their debilitating injury.

**Table-I:** Sociodemographic and clinical characteristics of patient with surgical intervention after lower limb trauma.

	n(50)	%
*Gender*		
Male	34	68
Female	16	32
*Socioeconomic Profile*		
Below poverty line	20	40
Above poverty line	30	60
*Parental Education (at least one)*		
Uneducated	11	22
Primary (grade 1-5)	9	18
Middle (grade 6-8)	7	14
Higher (grade9-10)	14	28
Intermediate (grade 11-12)	2	4
University (undergraduate/graduate)	7	14
	*Mean ± SD*	*Range*
Current age (years)	8.44 ± 2.14	5 – 12
Injury Severity Scores		
NISSSA	2.96 ± 1.16	0 - 6
GHOIS	4.24 ± 1.32	1 - 7
PTS	9.10 ± 1.36	6 - 12
Duration of Hospital Stay	17.94 ± 13.01	1 – 55

*NISSSA, Nerve injury, ischemia, soft tissue, skeletal injury, shock, age; *GHOISS, The Ganga Hospital Injury Severity Score; *PTS, Pediatric trauma score.

**Table-II:** HRQOL of patients undergoing surgical intervention after lower limb trauma.

Dimensions	Baseline		Follow-up		ES ^b^

	Mean ± SD	95% CI	Mean ± SD	95% CI	
*CHILD REPORT*					
Physical*	30.31 ± 21.71	24.15-36.48	86.13 ± 11.08	82.98-89.28	2.57
Emotional*	74.53 ± 17.83	69.47-79.60	85.30 ± 7.10	83.28-87.32	0.58
Social*	71.80 ± 13.99	67.82-77.78	93.10 ± 7.13	91.07-95.13	1.47
School^a^	---	---	89.74 ± 7.07	87.45-92.03	---
Psychosocial*	61.68 ± 12.13	58.24 -65.13	89.38 ± 4.46	88.12-90.65	2.24
Total HRQOL*	48.91 ± 13.95	44.94-52.87	88.04 ± 6.29	86.26-89.83	2.76
*PARENT REPORT*					
Physical*	23.93 ± 18.23	18.75-29.11	84.51 ± 12.80	80.87-88.15	3.34
Emotional*	68.60 ± 22.43	62.23-74.97	85.90 ± 6.12	84.16-87.64	0.71
Social*	72.28 ± 14.89	68.04-76.51	91.30 ± 8.01	89.02-93.58	1.20
School ^a^	---	---	88.72 ± 12.18	84.77-92.67	---
Psychosocial*	59.19 ± 14.05	55.20-63.18	88.58 ± 5.71	86.96-90.21	2.00
Total HRQOL*	44.96 ± 14.36	40.88-49.04	86.95 ± 7.53	84.82-89.09	2.93

HRQOL, health related quality of life

* p < 0.001

a baseline data for the school functioning domain wasn’t obtained as all the patients were not attending school owing to their debilitating injury.

b ES effect size measured as Cohen’s d: Calculated as mean change in score divided by the standard deviation of the difference scores. Effect size for differences in scale score means are designated as small (0.20), medium (0.50), and large (0.80).

Baseline HRQOL scores were significantly lower in patients and parent reported data taken at one week after surgery, with difference of 39.13 in total HRQOL scores between baseline and follow-up scores of the patients (p-value <0.001, overall effect size d=2.76), and difference of total HRQOL 39.86 between baseline and follow-up data taken from parents (p-value <0.001, overall d=2.93). The comparison of HRQOL scores of patients at six months follow up and their age-matched controls is given in Table-III. There was a clinically significant difference of 10.07 between the total HRQOL scores of patients (follow up) and controls and were higher than controls across all domains. In addition, parent reported scores were less than those reported by children in almost all cases (baseline, follow-up, and controls).

**Table-III:** HRQOL of patients (on follow-up) and age matched controls.

Dimensions	Patients (follow up)		Age matched controls		ES ^a^

	Mean ± SD	95% CI	Mean ± SD	95% CI	
*CHILD REPORT*					
Physical	86.13 ± 11.08	82.98-89.28	82.12 ± 12.20	78.66-85.59	0.34
Emotional**	85.30 ± 7.10	83.28-87.32	69.20 ± 21.10	63.20-75.20	1.02
Social*	93.10 ± 7.13	91.07-95.13	85.60 ± 17.71	80.57-90.63	0.56
School**	89.74 ± 7.07	87.45-92.03	72.80 ± 17.82	67.74-77.86	1.20
Psychosocial**	89.38 ± 4.46	88.12-90.65	75.62 ± 16.29	70.99-80.25	1.15
Total HRQOL**	88.04 ± 6.29	86.26-89.83	77.97 ± 13.69	74.08-81.86	0.95
*PARENT REPORT*					
Physical*	84.51 ± 12.80	80.87-88.15	77.56 ± 19.08	72.14-82.99	0.43
Emotional**	85.90 ± 6.12	84.16-87.64	75.60 ± 19.32	70.11-81.09	0.72
Social**	91.30 ± 8.01	89.02-93.58	79.00 ± 20.90	73.06-84.94	0.78
School**	88.72 ± 12.18	84.77-92.67	68.43 ± 22.61	62.00-74.85	1.08
Psychosocial**	88.58 ± 5.71	86.96-90.21	74.38 ± 17.46	69.41-79.34	1.09
Total HRQOL**	86.95 ± 7.53	84.82-89.09	75.49 ± 16.49	70.80-80.17	0.89

HRQOL, health related quality of life

* p < 0.05

** p < 0.001

a ES effect size measured as Cohen’s d. Effect size for differences in scale score means are designated as small (0.20), medium (0.50), and large (0.80).

There was no statistically significant relation between any clinical predictor/ sociodemographic data and difference of mean HRQOL scores. However, socioeconomic profile had an apparently significant relation (B=-8.37, 95% CI -22.19 to 5.46) with difference of mean HRQOL scores. Pediatric trauma score (PTS) was seen as a more consistent predictor of HRQOL difference, as compared to other clinical predictors, though statistically non-significant.

## DISCUSSION

This is the first study exploring HRQOL in children with trauma in an LMIC and was carried out in two major tertiary care hospitals of Lahore, Pakistan. This study also provides HRQOL scores of normal healthy school-going children in the local setting, which were not previously available. Patients were followed from discharge till six months and input was taken from parents and children. Results showed that lower limb trauma was associated with severe impairment of HRQOL scores in all domains especially the physical functioning.

Parent reported scores were also consistent with these findings (d=3.34). It was not surprising to find low HRQOL scores earlier as the impact of soft tissue injury and lower limbs fractures resulted in difficulties in mobility, and ability to do daily activities. Also, children with severe lower limb trauma had poor psychosocial outcomes (overall d 2.76 patients reported, and d 2.93 parents reported where d more than 0.8 is taken as significant), due to stresses resulting from the traumatic experience, hospital admission, surgeries, and changes in lifestyle. Several other studies also support the interaction between physical functioning affecting psychosocial quality of life and resulting in low academic performance eventually.^12^-^15^

Parent reported scores were less than child reported HRQOL, a finding that is consistent across studies comparing self-reports and parent reports for PedsQL.^16^-^18^ However, there was very small difference between parent reported and child reported QOL scores at six months’ follow-up (d 0.89 vs 0.95). This likely implied more awareness of their children’s health situation. Drastic improvement was noted in the HRQOL scores at time of follow-up suggesting faster recovery. This rapid recovery in follow-up is in line with findings of other studies.^19^,^20^ Another interesting finding was that the patients’ and their parents’ HRQOL ratings at follow-up outperformed the control group. This conclusion contradicts comparable studies,^20^-^22^ in which patients’ HRQOL did not recover to normal even after a time of up to 12 months.

This can partially be explained by selection criteria, since none of the patients included in our study had undergone amputation, or other forms of invasive procedures.^21^ Another explanation of these high scores could be that after a long period of suffering, pain and bedrest when the children were finally mobilized and able to do their daily chores and activities, there was an element of enhanced perception of well-being, concerning the biopsychosocial mode once more.

Avulsions and fractures were the predominant type of injuries with motorcycle spoke wheel injuries accounting for about half of all cases. Naumeri et al^23^ reported the increasing incidence of spoke wheel induced injuries in children in our study setting, supporting this finding. A higher percentage of participants were boys. As evident from surveillance of injuries in Pakistan, there was high prevalence of unintentional falls in males as compared to females of the same age.^24^ However, no sociodemographic variable had any impact on the QOL scores as shown by multiple regression analysis.

The only injury severity index that had a consistently negative association, although not statistically significant, with HRQOL scores was pediatric trauma score (PTS), meaning that children with lower PTS scores had poorer outcomes as compared to those with higher scores. Since the other injury severity scales, NISSSA and GHOISS, are not specifically designed for the pediatric age group, it can explain the absence of statistical impact of these scores on HRQOL. Winthrop A et al^20^ found correlation of injury severity score (ISS) with the QOL scores of children who suffered from trauma, but this relation was only reported in children less than five years of age. They also used different study tools namely Child Health Questionnaire (CHQ). Batailler, P et al^25^ also reported no association of gender, age, family socio-occupational level or other injuries to HRQOL scores but they also used CHQ for assessment of QOL scores.

### Limitations:

There were some limitations to this study. To avoid ethical issues, patients were not randomized and received different surgical treatments. Secondly, the sample size is small for associational analysis. HRQOL data from controls showed a large SD, warranting calculation from a larger sample size. Lastly, as some data was collected over the telephone, there might have been an element of miscommunication and lack of proper understanding on the part of the interviewer as well as subjects.

## CONCLUSION

HRQOL of children at discharge after management of lower limb trauma was lower than age-matched control group. It, however, significantly exceeded controls’ scores after six months showing complete recovery.

### Authors Contribution:

**MMH:** Collected data, prepared synopsis, approved the final version.

**MRA**: Collected data, took permissions from institutes/ Mapi trust, approved the final version.

**FN**: Conceived, critically appraised manuscript, approved and is accountable for manuscript.

**MMH**: Analyzed data, wrote draft, approved the final version.
